# How Does Being Solo in Nature Affect Well-Being? Evidence from Norway, Germany and New Zealand

**DOI:** 10.3390/ijerph18157897

**Published:** 2021-07-26

**Authors:** Evi Petersen, Annette Bischoff, Gunnar Liedtke, Andrew J. Martin

**Affiliations:** 1 Institute of Sports, Physical Education and Outdoor Life, University of South-Eastern Norway, 3800 Bø i Telemark, Norway; annette.bischoff@usn.no; 2 Department of Human Movement Science, University of Hamburg, 20148 Hamburg, Germany; gunnar.liedtke@uni-hamburg.de; 3 School of Sport, Exercise and Nutrition, Massey University, Palmerston North 4442, New Zealand; a.j.martin@massey.ac.nz

**Keywords:** wilderness solo, being in nature, solitude, emotions, well-being, culture, green exercise, nature connectedness, flourishing, PERMA-V

## Abstract

Background: Solo—being intentionally solitary in nature—is receiving growing attention as a valuable outdoor education program component. Its practice and history have been researched in the context of experiential learning, but few studies have explicitly examined how solo experiences can affect dimensions of well-being. This study investigated a broad range of well-being pathways provided by being solo, based on data from Norway, Germany, and New Zealand. Methods: Using qualitative content analysis (QCA), the solo debrief responses of 40 participants (26 females, age: 19–64 years) were analysed, applying the PERMA-V framework (emotions, engagement, relationship, meaning, achievement, and vitality). Variations in the reports were explored as a function of the national sample, gender, age, prior solo experiences and expectations. Results: The study suggests that hedonic and eudemonic well-being pathways, represented by the six PERMA-V pillars, interrelate strongly. The experience of a range of positive emotions and connecting process during solo highlights two of the most frequent findings related to well-being pathways. The secondary findings suggest minor variations in the well-being pathways for the different national samples, gender and age. Expectations and prior experiences with solo were identified as context factors with minor impact. Further, the data-driven analysis identified specific physical activities, landscape features, sense-activation, perception of time and ‘good’ weather as relevant to the specific experience. Conclusions: Solo experiences provide for well-being-related pathways in a multitude of ways, which highlights the well-being potential of solo implementation across practical fields beyond outdoor education, such as wilderness therapy, and environmental and planetary health initiatives. Future studies should continue to explore solo’s well-being potential in different settings, especially in the context of non-Western samples.

## 1. Introduction

*“To spend a lengthy period alone in the forests or mountains, a period of coming to terms with the solitude and non-humanity of nature is to discover who, or what, one really is–a discovery hardly possible while the community is telling you what you are, or ought to be.”*

(Alan Watts)

Most people living in contemporary Western societies have probably never experienced total social isolation for more than a day. Even in times of a global pandemic, many stay connected and receive social input through various digital channels. Taking into account that solitary confinement is considered one of the harshest punishments, one may wonder why an individual would intentionally choose to spend time in isolation.

Yet, the appreciation for solitude in nature as a way to facilitate personal growth, displayed in Watts’ poem above, is known to be historically rooted across many cultures [1]. *Wilderness solo, outdoor solo*, *nature-based solo*, or just *solo* are expressions to describe the specific body-based practice with the intentional goal of being in solitude.

Today, solo is an established component of contemporary outdoor or wilderness education and adventure programs worldwide, where it is typically practised as a facilitated experience, lasting approximately 24–72 h. Solo often emerges as one of the most impactful parts of outdoor programs for a variety of reasons. Focusing on the educational value of solitude in nature, research on solo has primarily evolved in the context of experiential learning. Theoretical understanding of the experiential learning process has been based on the work of Dewey (1938), most commonly described as a ‘cycle’ (Kolb, 1984) involving action, reflection and the transfer of learning [2]. In this scope, several studies have identified the phenomenon’s outcomes concerning personal growth (for a comprehensive overview, see [3]).

### 1.1. Solo and (Subjective) Well-Being

Some personal growth outcomes, such as introspection and mental clarity, e.g., [4,5,6], as well as self-actualization and reflections on meaning in life [7,8,9] can also be categorised as aspects of well-being. Further, an accumulating body of literature suggests that solo experiences can also affect one’s general sense of well-being, e.g., [10], and emotional or physical restoration, e.g., [8,11,12]. Prior investigation into the relationship between solo experiences and well-being is limited to self-report and many studies have focused on younger adults or adolescents, and in the context of educational outdoor programs, e.g., [6,12,13]. While the majority of current findings show that solo is associated with various positive aspects of well-being, solo can also have negative implications for well-being. For instance, studying 48 h solo experiences of adolescents over four years, Maxted [13] found a number of experiences that related to the emotion of fear. Participants feared aspects in nature, unexpected encounters with other people, and being alone. Maxted concludes that romanticising longer solos as spiritual growth opportunities for everyone might be misleading.

While aspects of self-actualisation and situated emotional reactions, such as fear, seem to be disparate, they can be sorted into hedonic (e.g., the experience of pleasure) and eudemonic (e.g., the experience of meaning) dimensions of overall subjective well-being [14]. Subjective well-being is a complex phenomenon, which is why several decades of research in its scope has resulted in a plethora of measurement instruments and explanatory models [15]. According to Keyes [16], the concept of (human) flourishing unites a broad range of different subjective well-being aspects, such as emotional well-being, psychological well-being, and social well-being. In this context of positive psychology, Seligman [17] has proposed the PERMA model to capture overall well-being. This model builds on five pillars, of which some pillars target hedonic, and others target eudemonic aspects of well-being: ***P****ositive emotions*, ***E****ngagement*, ***R****elationships, **M**eaning, **A**ccomplishment*. Seligman suggests these pillars to be important distinct areas that people pursue for their own sake. By adding a body-based component, the ***V****itality* pillar, PERMA-V has emerged as an extended version of the model. According to Eacker [18], the V of the extended version has been proposed by Zhivotovskaya, from the Flourishing Center, in 2014 to critique the absence of a body-related measurement in the original PERMA.

No empirical investigations of the PERMA-V model in the context of solo or within the scope of outdoor research can be found in the literature to date. However, Yerbury and Boyd [19] applied the basic PERMA model to well-being-related outcomes of human–dolphin interactions. The authors suggest that people’s interactions with wild animals in their natural settings may uniquely contribute to well-being and connection to nature.

In sum, little is known about how or through which pathways solo affects well-being. This is surprising since solo, as a contrasting experience to daily life, has an anecdotal reputation for enhancing the quality of participants’ experiences on a physical, mental, and emotional scale [12].

Moreover, Tam and Milfont [20] persuasively argue that cultural conceptions of nature enable, limit, and shape nature experiences. This argument has also explicitly been stressed for social relational emotions elicited by interactions with nature [21,22]. Grasping such cultural situatedness is of particular importance for solo settings, since the practice of being solitary as an end in itself is historically and culturally rooted.

### 1.2. Solo’s Context

Although the broader question about the suite of well-being impacts gained from solo has only fractionally been addressed, focused explorations have investigated some of solo’s essential context variables. The primary discussed context factors of solo experiences are those targeting pedagogical framing and facilitation. In this scope, the role of the instructor as an essential facilitator for the learning experiences has traditionally been stressed by several studies [23,24,25]. Williams [26], however, argues that self-facilitated solos might be as effective concerning learning outcomes as long as participants are provided with the possibility to stay physically comfortable when facing new or uncertain demands experienced on a solo.

While pre-experience, expectations and personal preferences have been suggested to be elements affecting the perceived outcomes of the solo experience [27,28,29], solo experiences also vary according to several demographic factors. For instance, gender and age have been investigated, showing complex or inconsistent relations regarding solo experiences [9]. Because of these interactions, the well-being impact afforded by solo time spent in nature is examined according to gender and age in the current study.

Knapp and Smith [1] also noticed that those who have spent several days or weeks in solitude report particularly meaningful and memorable experiences. This prompts the questions of whether and how the impact of solo depends on its duration.

Moreover, solo has been performed in a variety of nature types such as mountains, e.g., [29], coast or lake landscapes, e.g., [12], wooden areas, e.g., [30] and even in urban nature, e.g., [31]. While recent findings suggest that different types and quality of environment have different psychological benefits associated with them [32], no scientific investigation has yet empirically compared the impact across specific types of nature environment on solo experiences. In this context, other situational context factors such as the weather have been repeatedly mentioned by participants [29] and, therefore, qualify as influential factors on the solo experience that are worth investigating further.

### 1.3. Research Objective

Uncovering the range of well-being pathways may enable the use of solo practice as a resource in various practical applications regarding health maintenance, wilderness therapy, and environmental and planetary health promotion. Given the limited number of studies on solo and well-being, the broad scope and limited integration to date, the present study is designed to identify and organise the potential pathways of solo experiences leading to well-being within a comprehensive framework.

The present study’s primary objective was to explore the self-reported well-being-related impact of solo experiences by applying the PERMA-V framework. A secondary objective was to examine the variations of these experienced impacts according to influencing contextual factors such as socio-cultural demographics, experiences and expectations. This study uses a qualitative method approach, which is informed by a postpositivist interpretive framework [33], and may be placed within the field of outdoor studies, drawing on theoretical conceptualisation provided by environmental and positive psychology. It focuses on the age group of younger and middle-aged adults in contrast to many other existing studies exploring solo experiences in youth. Further, most solo research has focused on specific program-based outdoor education providers in the United States. The present study explores solo across different educational settings in two European countries and New Zealand.

## 2. Materials and Methods

### 2.1. Recruitment and Participants

The three different national samples of participants were recruited through typical case sampling, a type of purposive sampling, useful when studying a phenomenon related to those considered average members of the affected population [34]. The decision to study these three samples was based on the established routines of solo implementations within the institution or organisation and a shared interest in the growing discourse targeting nature-based interventions to improve human and planetary well-being. The first author reached out to the specific institutions hosting the solo events and asked for permission to approach the target groups. After permission was granted, participants of all three samples were informed about the study and asked for voluntary participation. To be included in this study, participants had to be 18 years of age or older. Participants were 42 adults from Norway, Germany and New Zealand (26 females, age: 21–64 years; Mnd = 30; SD = 10,33). Two participants did not wish to be included in this study due to personal reasons. The final sample size of 40 participants is larger than other solo studies that have qualitatively analysed participant-generated data of adults, e.g., [5,7,9,10]. The overall sample size of this study was determined by data saturation in order for the results to be capable of some degree of generalisation [35]. According to Mayring [36], the sample size of this study is considered appropriate, given that the primary purpose of this explorative study was to develop a depth of understanding, and sampling for proportionality not being the primary concern. The recommendation of Guest, Bunce [37] and Boddy [35] to include a minimum of twelve in-depth interviews for each sample population in qualitative research was followed. A difference to be noticed is that while the German and Norwegian samples consisted of university students, the sample from New Zealand presented itself with diverse occupational backgrounds. A comprehensive overview of the participants’ characteristics is displayed in Table 1.

### 2.2. Solo Contexts and Procedures

All three solo implementations were group-based and embedded in a larger course setting, consisting of different outdoor-based activities. All participants were advised to bring as little equipment to the solo trip as possible. Moreover, participants were not supposed to bring alcohol, tobacco or use technical devices during the trips. Table 1 also provides a visual overview of the research design and the specific context of experience. In each sample, during the briefing on the day before the solo started, participants filled out a questionnaire focused on pre-experiences with solo and their expectations. The interview material was gathered during the debriefing on the day the solo had ended. The term debriefing is typically used in the context of facilitating experiential learning. The overall purpose of debriefing is to help the participant reflect on the experience, transfer what is learned from one setting to another, and deepen their understanding of what they have learned [38]. The debrief situations of the three national samples were similar to each other, immediately performed after the solo, close to the overall solo area, each session lasting approximately two hours. Gathered in a group, participants individually shared their experiences. It was stressed by all instructors that everyone could share precisely the sort and amount of information that they felt comfortable with. There was an open discussion of general questions and tasks in the debrief closing, which are not subject to this study.

### 2.3. Measures

Participants talked about the following aspects of their experiences in the debrief situation: (1) Description of the place in detail. *Where did you go? What was it like there? What were the features of our surroundings?* (2) Description of their activities. *What did you do? (*3) Description of the perceived experiences. *How did you experience your solo time? How did you feel, what did you think about?* Additional concept-driven context variables (conducted through a pre-solo paper and pencil questionnaire) were the following:

- Socio-demographic information: participants reported their age and gender.
- Prior experiences with solo and outdoor activities (yes; no).
- Expectations about the solo (mainly positive, mixed, mainly negative).

The participants also completed an additional survey with closed-ended questions before and after their solo experiences. These data were used to investigate a separate research question in another study, so we do not report them here.

### 2.4. Data Analysis

**Figure 1:**
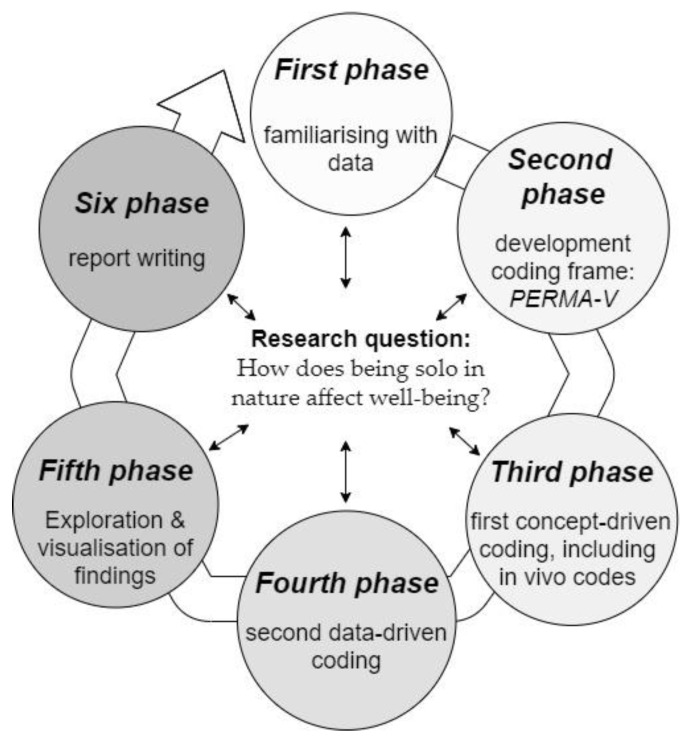
The six phases of the qualitative content data analysis, adopted from Rädiker and Kuckartz [41].

**Figure A1:**
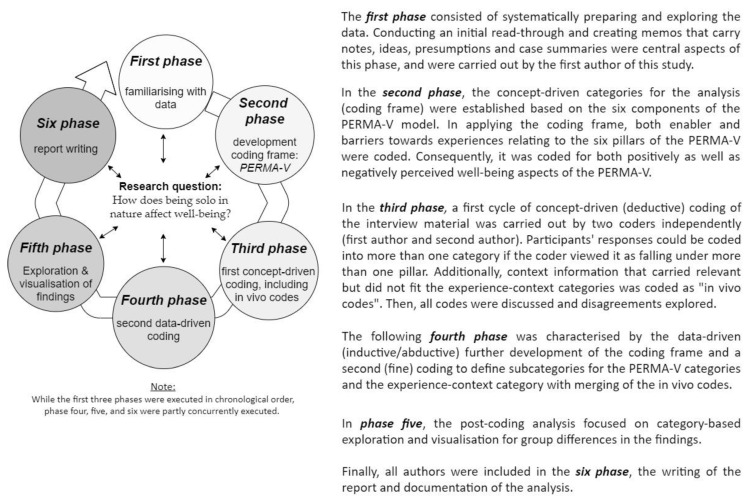
An additional figure comprises a detailed description of the six steps of the data analysis.

For this study, a qualitative content analysis (QCA) was applied. QCA is a rule-guided method for systematically describing qualitative data’s meaning [39,40]. QCA can be used to summarise a huge amount of information by assigning successive parts of the material to the categories of a coding frame. This approach focuses on selected aspects of meaning, namely those related to the overall research question. The leading question for the present study was: “What are the pathways through which solo may affect well-being?” The data analysis followed the procedure proposed by Rädiker and Kuckartz [41] for the systematic and focused analysis of qualitative interviews. This step-by-step process model builds on several established QCA and other approaches for analysing qualitative data, such as those proposed by Mayring [42], Schreier [43], and Creswell [44]. The analysis procedure consisted of six phases executed in MAXQDA (version 20.4.0), an established software for qualitative data analysis. While the first three phases were executed in chronological order, phase four, five, and six were handled concurrently. Figure 1 provides a brief visual impression of the data analysis procedure, and an additional figure (Figure A1 in the Appendix A) comprises a detailed description of the six steps of the data analysis.

The six components of the PERMA-V model were chosen as an initial coding frame to explore the different pathways through which solo experiences may affect well-being in the context of solo. The model is rooted in research data collected within Western, Educated, Industrialized, Rich, and Democratic (WEIRD) cultures. The present study is situated in three different national settings (Norway, Germany, and New Zealand), which all fall under the WEIRD definition [20]. Consequently, PERMA-V was considered appropriate scaffolding for the data of this study. It was coded for both positively and negatively perceived aspects of the well-being pathways. PERMA-V consists of six components: (1) ***P****ositive emotions* refer to the hedonic aspects of well-being (e.g., feeling cheerful, happy, grateful). (2) ***E****ngagement* refers to a psychological connection to activities (e.g., feeling absorbed, interested, and engaged in life) and is related to the concept of flow [45,46]. (3) ***R****elationships* include feeling socially integrated and being satisfied with one’s social connections. (4) ***M****eaning* refers to believing that one’s life is valuable and feeling connected to something greater than oneself. (5) ***A****ccomplishment* involves making progress towards goals and feeling capable of undertaking daily activities. The additional V stands for (6*) **V**itality* and includes physical restorative aspects such as sleep, nutrition, and physical activity.

Comparative research across cultures and nations provides challenges concerning the standardisation of data collection and analysis [47]. Concerning the cross-national nature of the current study, the content analysis proceedings, and in particular, the coding process of the present study, are in line with the principles of openness, context-sensitivity and multiperspectivity as proposed by [48] in the context of cultural qualitative content analysis.

For the purpose of improving the rigour and sincerity of qualitative inquiry [49], it should be reported that the first author’s personal experiences with solo, on her own and with groups, led to an interest in conducting this research. The first author conducted, audio-recorded, and transcribed verbatim all data in the native tongue of the participants (Norwegian, German or English). Quotes from the Norwegian and German interviews used in the results section were subsequently translated into English. Independently of the original language, some quotes were shortened for reasons of readability. The first and second author carried out coding of the interview material independently. An intercoder agreement coefficient of 0.93 for analysis of code frequency was calculated, which attests to the robustness of the coding process [50].

Lastly, it should be acknowledged that all authors of the present study are working within the field of outdoor education and have years of experience with solo practice in at least one of the cultural contexts researched in this study. Thus, the author’s personal and professional experiences with solo necessarily influenced the data analysis and the research findings.

## 3. Results

### 3.1. PERMA-V

**Figure 2:**
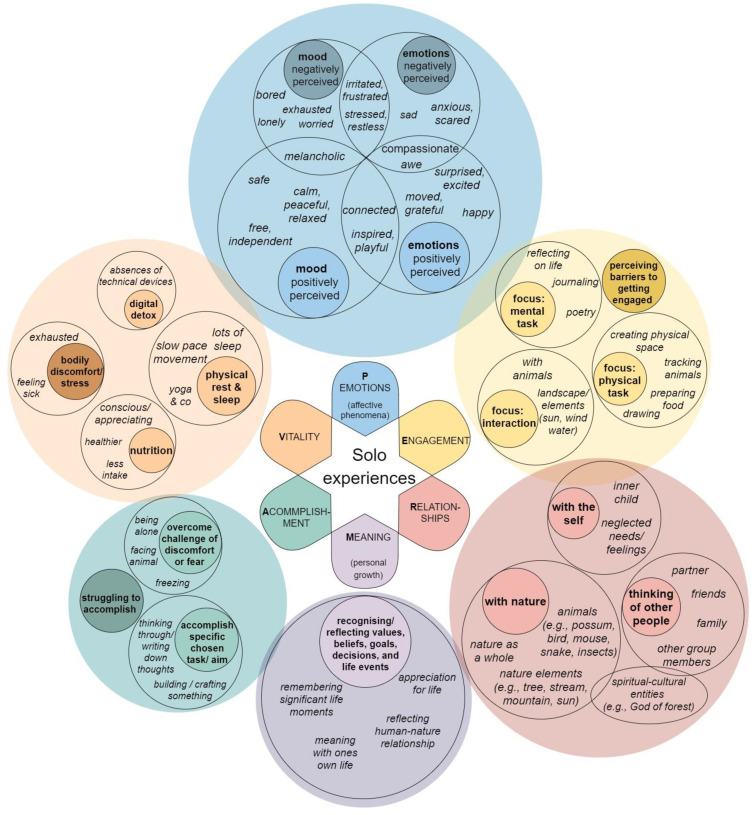
Well-being pathways, applying PERMA-V: (Positive) emotions, Engagement, Relationship, Meaning, Accomplishment and Vitality, including subcategories. The relative visual size of the circles at each level reflects the relative frequency of segments coded into each category.

Figure 2 displays the six investigated well-being pathways related to PERMA-V: (Positive) emotions, Engagement, Relationship, Meaning, Accomplishment and Vitality, including frequency for each category. For the combined sample, the categories and their subcategories are described in detail below. Participants’ quotes are presented for exemplification. The overall percentage of participants who reported the category at least once is presented in addition to the frequency of coded segments in the debrief transcripts. Overall, the frequency of coded segments for well-being aspects expressed per participants ranged from zero to nine (M = 1.7; SD = 1.6).

#### 3.1.1. Emotions and Mood (Affective Phenomena)

Except for one, all participants mentioned affective phenomena at least once (144 coded segments), which were divided into emotions (short-lived, potential to be strongly experienced) and mood (relatively long-lasting states, often more moderate or light in experienced intensity).

In total, 75 coded segments presented mood descriptions, of which 83% were perceived as positive. The most frequently mentioned positively perceived mood descriptions were feeling calm, peaceful, and relaxed. For example, “It was absolutely peaceful”, and “I have enjoyed myself these days, like I was relaxed, went on summit hikes, slept a little and slept a little more, wrote a poem and sang a few songs, and watched the sunset”). There were also expressions of feeling free and independent, such as “… it was pleasant because I knew, I have a lot of time and a lot of freedom. I can stay where I am but I can also go into five different directions”. Feeling safe was also noted: “I created a space for myself, in which I just felt safe”. However, six participants also reported negatively perceived moods such as being worried or feeling exhausted. For example, “I was worried that someone would find me sleeping there” and “the next day, I was exhausted from not sleeping well during the night”. Feeling bored and lonely was also reported: “I don’t like being by myself for too long, I guess, it is just so boring “and “I realised, now I’m alone, I have to do everything myself, so that took a bit away the joy”.

There were 69 segments coded in the emotion subcategory, of which 75% were positively perceived. Overall, the most frequent responses were expressions of surprise and excitement: “seeing the glow worms felt amazing because it was a surprise”, and happiness: “I had this moment in which I just felt incredibly happy”. Social relational emotions such as gratitude and being moved (≈ kama muta) were also frequently noted. For example, “I noticed that I’ve become much more grateful for the small things in life” and “I could see everywhere, it is absolutely beautiful. I was so touched [rørt] by the connectedness to nature [naturnærvær]”). In contrast, 36% of the coded segments were reported as negatively perceived emotions such as sadness: “it really made me sad to see all these trees having being cut down”, and anxiety: “I have to admit, I was quite scared of the possums, especially the first night”.

Most descriptions could be placed into either the emotion or mood subcategory, while some descriptions clearly belong to both categories (11%). The overlap was widespread for descriptions of feeling connected: “I always feel connected when I’m in the bush, it is just that in some moments you are more aware of it”. Moreover, feeling inspired and playful was placed into both emotions and mood categories: “So inspiring, it felt like being a child again, exploring the space around me, just lots of great moments”, as well as feeling frustrated, stressed and restless “I did not want to just think about everything, that was when I got a bit restless”. A categorical overlap was also evident for descriptions falling in between being positively or negatively perceived, such as compassion, awe, and melancholy. For example, “the view was stunning and I got this feeling of real fascination but it also a little weird because you realise how small and insignificant you are” and “I quickly entered a melancholic state, reflecting my current life situation, which kind of felt good and bad”.

#### 3.1.2. Relationships

With 93 coded segments, the second most frequently mentioned well-being pathway was that of relationships, recognised at least once by 95% of the participants. Relationships descriptions were divided into three subthemes: relation to nature, relation to other humans and relation to the self. Descriptions of relationships to nature were most frequently reported (65 coded segments), and often included experiences of connecting to animals. Most encounters were positivity perceived, “the most positive experience for me on this trip must have been the closeness [nærhet] to the grouses”; however, a few participants also experienced encounters with animals as challenging, “I had a feeling that someone was staring at me, and when I look behind me there was this viper… I took the world record in packing my things… I quite panicked”. Other participants described some relationship to abstract nature, and three participants mentioned their relation to spiritual and cultural entities. For example, “Tāne is the God of the forest and the birds are his children. So when the birds come around, I just sort of think, well, that’s one of his children”. A total of 22 participants described thinking about their relationship to others, including their partner, family, and friends: “I was reflecting on things, like I thought of people close to me and my relationship to them”. A few people also mentioned relationships to or within themselves:

I was kind of debating with myself… it became clear to me that I have a little conflict within me regarding my feelings. On the one hand, I need security in order to feel good, otherwise, I somehow have a bad feeling in my stomach… on the other hand, I also somehow have the feeling or need of freedom, because I often feel twitchy in the city (female, 26 years, Germany).

#### 3.1.3. Engagement

Aspects of engagement were reported by 85% of the participants (60 coded segments). Most participants reported been engaged in the activity of interaction (34 coded segments), such as with elements of nature and the landscape, as described by a 46-year-old female from New Zealand:

I really did think I probably would be sitting there reflecting about my life and my future but I honestly didn’t feel like doing that. I was happy just looking at the plants and birds and like just enjoying being there in that spot, which is quite unusual for me (…) I really felt like the experience was beneficial for living in the moment.

Some participants (20 coded segments) were focused on a physical task (e.g., preparing food), and others (16 coded segments) reported being focused on a mental task (e.g., poetry). Moreover, six participants reported perceived barriers that prevented them from moving into a state of engagement. Besides missing equipment and disliking the silence, the most commonly reported reason was lack of physical movement: “(…) normally I am more physical and motion helps me think, too. So maybe sitting still is a time to recharge, but also the time that the brain activity just goes like at rest time”.

#### 3.1.4. Vitality

About 83% of all participants (62 coded segments) reported some form of physical vitality. Most participants talked about the opportunity for bodily recovery through physical rest and getting more sleep compared to their daily life: “My intention was just to enjoy myself, relax and catch up on sleep… and I did that, which was awesome”. A few participants reported engagements in specific forms of low-intensity physical exercise, such as yoga: “In the morning, I’ll do a ritual of like five sun salutations”. Moreover, six participants noticed aspects of their nutrition behavior, such as more conscious consumption: “I was paying attention to my body, waited to cook until I got really hungry”. Three participants talked about a perceived digital ‘detox’ effect. For example, “I think it was really nice to experience being completely alone and not having a watch or a mobile phone”. However, five of the participants also reported bodily discomfort such as feeling exhausted, and two additional people reporting having felt sick: “The only thing I struggled with was the sleeping on the mat, it was quite uncomfortable” and “I fell asleep in the sun and I felt really sick afterwards”.

#### 3.1.5. Accomplishment

Accomplishing a specific task or overcoming the challenge of discomfort or fear were mentioned by 66% of all participants (34 coded segments). While 3 participants noted having struggled with accomplishing a goal, 22 participants reported successful accomplishments. For example, “I’m normally really scared to be alone, but I found that it was actually not too bad to be alone. It felt good to overcome that fear”, and “Being in that spot helped me to overcome my writing issues”. Similar to the category of meaning, participants stressed these aspects of experience as valuable, exemplified in a quote of a female 61-year-old from New Zealand:

That shift in perception from thinking that the possums might harm me to ‘the possums are just looking for food’ was really helpful. It was positive and felt really nice to eradicate that fear, just to think, ‘I can do this again, this is okay’.

#### 3.1.6. Meaning

Aspects coded as meaning were reported by 53% of the participants (36 coded segments). Most participants described a form of recognition or reflection on their values, beliefs, goals, or decisions. Herein, one-third of these participants who mentioned meaning specifically emphasized some sort of appreciation for life. For instance, a 28-year-old female from Norway expressed appreciation for the lived moment in the context of viewing a nature landscape from the top of a mountain: “There was this really nice view and I got this ‘wow, is that possible’-feeling. I had to sit there for a while. I felt like a king or queen in that moment”. Reflecting on the relationship of humans to nature was a specific topic for eight of the participants:

It is important for us humans to engage in sustainability because we can influence nature to such a large extent, even if that does not mean that we control nature. I really believe that we are only a small part of nature that, somehow, has more influence than other parts of nature (male, 27 years, Germany).

Furthermore, meaning also expressed itself as remembering significant life moments (five participants, six coded segments). One participant reflected on having moved to another city “I thought a lot about that decision and if it was the right call to make”. In this scope, four participants also reported thoughts connected to their life situation and future plans: “This question, what will be in a few years or so, was omnipresent for me”.

### 3.2. The Interrelation of the Well-Being Pillars

**Figure 3:**
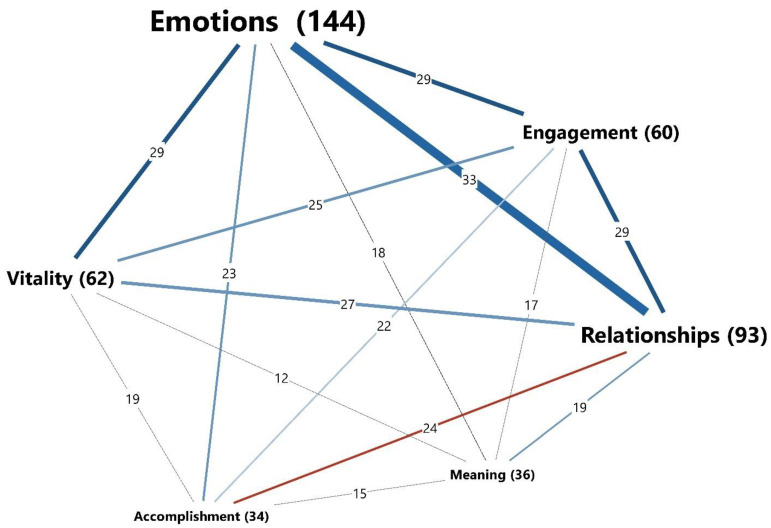
The six main categories and the strengths of their connectivity based on the frequency of the coded segments in the debrief transcripts (presented in parentheses). The feature Code Proximity model of MaxMaps in MAXQDA was used to generate the visualisation.

As displayed in Figure 3, all six main categories are, to varying degrees, interrelated. While the category of emotions has the most established connections to the other well-being pathways, its most frequent link is that to relationships. This proximity or overlapping of the emotions and relationships categories is illustrated in the following quote from a female participant (21 years) from Norway: “I think I was actually quite moved [rørt] when I sat down and thought about all the great people around me on a daily basis… and how lucky I am”. However, relative to the frequency of all codes for each category, the strongest links were those between the categories of accomplishment and meaning to the other four categories. For instance, the category of accomplishment has a total of 34 coded segments, of which 24 coded segments (71%) overlapped with the category of relationships. This quote from a female participant from Germany (29 years old) illustrates the accomplishment of a change in perception regarding her relationship to insects:

One more thing that struck me was that I developed a tolerance for crawly animals like beetles and spiders… I observed this caterpillar, which I usually found disgusting, and suddenly looked closer to see that it is just an animal, which moves very differently than me and has a completely different microcosm but in the end we both just live here on earth together. That totally changed my perspective and I am really happy about that.

Moreover, one participant recalled, “I wrote a couple of poems about what had happened to me in the past… bringing those emotions up kind of made me cry, it was really purifying and actually a quite cool experience”. This quote illustrates the interrelation between the category of meaning and emotions.

### 3.3. In Vivo Codes

Naturally, participants also mentioned some other aspects of their solo experiences that did not fit the PERMA-V coding framework. The following most frequently mentioned aspects were generated as in vivo context codes in the third and fourth phase of the data analysis: satisfaction with specific solo activities (mentioned by 26 participants), the appeal of specific landscape characteristics (mentioned by 15 participants), activation of the senses (mentioned by 13 participants), differences in perception of time (mentioned by 10 participants), and the importance of ‘good’ weather (mentioned by 9 participants).

### 3.4. Impact of Context Variables

An overview of the relation between the context factors and the six well-being pathways is provided in Table 2.

#### 3.4.1. Similarities and Differences in Themes between National Samples

Overall, all six main well-being pathways were present for all three national samples. Nevertheless, minor variations were noticed regarding the frequency of coding, especially at the subcode level. For instance, the category of meaning was most prominently mentioned by German participants, while descriptions of encounters with different types of spiritual entities were only found for the New Zealand sample. The latter sample was also the only one to report engagement barriers. The Norwegian sample showed the most considerable internal variations for the various well-being pathways.

#### 3.4.2. Demographics (Gender, Age), Prior Solo Experiences, and Expectations

The frequency of each of the six main categories was also examined by gender, age, pre-experiences and expectations. Female participants generally reported a broader range of well-being-related aspects than males. Females were also more likely to talk about negatively perceived emotion. Younger adults were slightly more likely to report eudemonic well-being aspects related to the category of meaning than middle-aged participants. In contrast, middle-aged adults reported, overall, more frequently positive perceived hedonic aspects compared to younger adults.

Participants that had been on a solo previously mentioned a range of hedonic well-being aspects. In contrast, aspects of eudemonic well-being related to accomplishment and meaning were less often reported among these participants.

Noticeably, none of the participants expected the solo to be an overall negative experience. Four participants, who had prior solo experiences, expected the solo to be a complete experience, including positive and negative experiences. All other participants expected the solo to be an overall positive experience.

## 4. Discussion

Despite growing research interest in the benefits of solo experiences, the current study is the first to propose a comprehensive and detailed exploration of the well-being pathways elicited by solo experiences. It validates and elaborates on some previously discovered well-being benefits of solo experiences, such as aspects of emotional and physical restoration, e.g., [8,11,12] as well as reflections on meaning in life [7,9]. Further, the current study explores how a broad range of self-reported well-being-related aspects vary across national samples, demographics, and context characteristics. By identifying which reported well-being aspects are universal and infrequent, this study offers a rich understanding of how people experience solo and how these experiences are linked to well-being.

### 4.1. A Multidimensional Well-Being Framework for Solo and the Interrelatedness of Its Categories

The six pillars of PERMA-V served as a multidimensional framework to explore the range of hedonic and eudemonic well-being pathways provided by solo. This study elaborates on the basic PERMA framework in two main ways. First, it extends the understanding of the psychological process dimension of the PERMA model by including a physical level of well-being (Vitality pillar). In line with the literature suggesting physical components to be a crucial part of well-being [51], different aspects of bodily vitality have been frequently coded in the present study. Yerbury and Boyd [19] applied the basic PERMA framework to investigate well-being effects elicited by human–dolphin interactions. Their study results also show that physical well-being-related aspects, such as heightened sense activation, were frequently mentioned by the participants. Second, expanding the framework to positively and negatively perceived experiences allowed for a comprehensive overview of solo’s possible impact on well-being. Overall, the elaborated PERMA-V framework proved to be a suitable approach for identifying and organising the potential well-being pathways for the three national samples of this study.

The interrelatedness of the different dimensions (Figure 3) suggests that solo is a complex phenomenon building on interrelated aspects of experiences. These findings give meaning in light of a large cross-cultural study [52] that found hedonic and eudemonic pathways to correlate significantly when predicting overall subjective well-being. Similar to the findings of Yerbury and Boyd [19], the interrelatedness of the categories—emotion and relationships—was found to be particularly frequent. However, the causality of this relationship can only be speculated. While previous research most often has viewed emotions and related affective phenomena as outcomes of contact with nature, e.g., [53], recent studies highlighted the role of social relational emotions in establishing and intensifying nature connectedness [21,22,54]. Moreover, based on a study with a representative sample from England, Martin et al. [55] found that nature connectedness was positively related to eudemonic well-being and pro-environmental behaviour. In sum, the complexity of well-being pathways and contact with nature is worth further investigation.

### 4.2. Hedonic Well-Being Pathways

Overall, hedonic well-being pathways, coded into emotions, engagement, and vitality, were the most frequently mentioned aspects of the solo experiences. It may not be surprising that hedonic aspects were more frequently coded than eudemonic ones, given that the latter demands more in-depth reflections of the participants.

The emotion pillar was the most frequently mentioned hedonic well-being pathway, including both positive and negative moods and emotions, as well as overlaps between those subcategories. Across all national samples, most participants experienced affective phenomena of feeling calm, peaceful and relaxed. These are findings that previous research has revealed for different participant populations [7,9,12]. The consistency of these findings suggests solo as a promising nature-based intervention for stress-related issues. Richardson [56] suggests a three-circle-based that explain the well-being benefits of nature through balancing emotion regulation. Interestingly and despite being alone, participants reported a range of positively perceived social relational emotions such as being moved (≈ kama muta), gratitude, awe, and compassion. These findings support a recent proposal of Petersen et al. [21] that social relational emotions underpin both social and nature connectedness.

In contrast, negatively perceived emotions during solo have often been neglected in previous studies. Nevertheless, Maxted [13] found loneliness and fear to be reoccurring and prominent topics among adolescents. Emotional descriptions of being scared or anxious were also reported by about 20% of the participants of the current study. However, only three participants mentioned some form of loneliness, which stresses the profound difference between being alone and feeling lonely. The socioemotional benefits of ageing [57] may explain some of the variations in findings to those of Maxted [13]. In particular, emotions such as melancholy, sadness and anger have rarely been reported in previous solo studies. The current study found that several participants reported negatively perceived emotions, feeding into the environmental anxiety discourse—a contemporary cross-cultural fear of destroying nature [58]. Those emotions were often linked to the motivation of engaging in pro-environmental initiatives and behaviours: “To hear few native birds made me sad… but it encourages me to do more, you know, get out there and help more with predator control”. One explanation for this finding could be that individuals who experience intense negative emotions hold strong motivations to regulate these [59]. Consequently, these findings suggest that negatively perceived emotions hold the potential to influence the contemporary discourse of planetary health in a proactive way. Overall, the complexity of findings in the emotion category suggests that emotions and other affective phenomena deserve greater attention within solo research.

The two other main categories for hedonic well-being pathways were engagement and vitality. Specifically, aspects of vitality in the form of relaxation have been frequently mentioned in previous solo research [8,11,12]. The findings of this study are novel because perceived well-being barriers, such as bodily discomfort for the vitality category and lack of physical movement for the engagement category, are reported. A better understanding of the perceived barriers can inform the successful facilitation of solo practice, particularly in the prevention and treatment of stress-related phenomena. One participant fittingly noted: “It was the kind of almost ultimate experience to make you relax and forget about everything else… I mean, some people go on spa for that”.

### 4.3. Eudemonic Well-Being Pathways

While previous solo research has mentioned some hedonic well-being aspects of solo experiences, most previous solo research has focused on aspects of eudemonic well-being. Knapp and Smith [1] stated that alone time in nature could create space for insights, inspiration, and connection to self and the broader community of life. The current findings support and expand on these eudemonic well-being pathways. Relationships with nature, with the self, and with others were the most frequently reported eudemonic well-being pathway. Similar results were found in a study by Williams [26] in the context of experiential learning. The author found that solo as a learning experience can help to transform a person’s view of themselves, their relationships with others, and their connection to the natural world. The well-being-related benefits of the relationship to animals have been particularly stressed in the human–dolphin interaction study by Yerbury and Boyd [19]. In the current study, relationships to various animals are reported, including those to insects. It has been argued that connecting to nature may be a basic psychological need and, thus, crucial to well-being [60,61].

Further, in the current study, connecting to the inner child and opening up for neglected feelings or needs described forms of connecting to the self. Previous solo studies have found similar solo outcomes [4,10]. Schuling et al. [62], for instance, noticed that participants reported compassion for themselves and others after a silent mindfulness walking retreat. In general, the idea of self-connection is a relatively new concept that is rooted in self-awareness, involving accepting and aligning behaviour based on that awareness [63]. Knapp and Smith [1] comment that solo could provide awareness, understanding, and clarification of one’s place, purpose, and direction in life. Thus, self-connection might be the most valuable well-being pathway that solo experiences can offer for practical applications focused on wilderness therapy.

Although the categories of meaning and accomplishment were less frequently reported, the participants of this study specifically expressed appreciation for experiences linked to these categories. The meaning category contained aspects related to the individual life and purpose, frequently mentioned in previous solo studies [24,26]. However, many participants also reported that they had reflected on the human–nature interaction, which stressed the contemporary awareness for societal issues such as environmental and planetary health, which should be followed up in future studies.

Lastly, accomplishment has often been a primary goal in old traditions of the practice in different cultural contexts, for instance, during a vision quest, which is a rite of passage in some Native American cultures. Although today’s solo challenges might be less dangerous, studies have mentioned this category as a beneficial solo outcome, especially in the context of experiential learning, e.g., [12].

### 4.4. Three Different National Samples

Although the three different national samples underwent solo under a range of similar context factors (e.g., voluntary; receiving briefings), they also differed regarding other contextual factors (pre-experiences with solo, age, duration of the solo, specific landscape, and whether they chose their specific solo spot). These circumstances make it impossible to draw firm conclusions from their comparison. However, the results open up for some profound discussions, stimulated by the idea that aspects of psychological phenomena such as subjective well-being could be universal and/or culture-specific informed. For one, solo is a practice that removes an individual from its social context. A universal question in the scope is: Why does the absence of the social factor affect well-being? Drawing from a phenomenological point of view that considers the corporal body, its senses and emotions, and the world around it, Frers [64] argues that the experience of absence could feel more substantial when it refers to practices, emotions and corporal attachments that have been deeply ingrained into those who experience the absence. For the solo experience, this could mean that the participants consciously or unconsciously tried to reduce human isolation by seeking out a connection with, for instance, animals and landscape elements. This, in turn, could lead to feelings of connectedness and the experience of valuable relationships with nature. Such argumentation would also be in line with the concept of resonance, recently proposed by the sociologist Rosa [65], after who’s theory of acceleration, solo time could be considered an opportunity for a time-out from the social pressures commonly felt in Western society.

Continuing with the example of connecting processes during solo, only participants from New Zealand reported connectedness to spiritual entities, for instance, Tāne, God of the forest. Here, Tam and Milfont [20] propose that humans are situated within layers of both place and culture, and that this cultural milieu, again, influences the physical reality of places. Hence, human–environment interactions can be considered culture-bound. Moreover, Miyamoto and Ma [66] provide evidence that cultural scripts guide the regulation of positive emotions towards amplifying or dampening the experienced emotion. In this sense, cultural scripts may influence how participants experienced their emotions and other aspects related to well-being pathways. Additionally, emotions dependent on the relational meaning that individuals construct from an interchange [67]. Thus, to better understand the cultural situatedness of solo experiences, the question of what it means to be a Norwegian, German, or New Zealander is as crucial as what the individual understands under the concept of nature.

### 4.5. Demographic Similarities and Differences

Overall, the self-reported well-being aspects appeared to vary little by gender and age. One of the minor gender-related differences was that women mentioned more well-being aspects overall than men did. They were also more likely to describe emotional experiences of fear and anxiety. The latter is consistent with the findings of Ryer et al. [68]. It might be that women feel more comfortable talking about private themes, or solo could trigger different gender-related challenges for females and males. Either way, the relationship between gender and well-being outcomes from solo is worth future in-depth exploration.

Moreover, the findings suggest that younger and middle-aged adults experience similar well-being pathways. Nevertheless, younger adults tended to more frequently report aspects related to the meaning category, while middle-aged adults more frequently reported overall positive perceived hedonic aspects. Kalisch et al. [12] found similar differences in reported solo benefits when comparing debrief responses of youth against those of younger adults. Developmental differences affecting the experience of different well-being aspects across different age groups are likely since humans face different kinds of life events and challenges throughout the lifespan. For instance, younger adults are more likely to search for purpose and personal growth, which is, indeed, more strongly related to meaning-related pathways to well-being [57]. However, the topic is potentially subject to future studies.

### 4.6. The Importance of Context Factors for the Facilitation of Solo Experiences

The context factors of the solo were derived from the questionnaire data before the solo, and the data-driven in vivo codes from the solo debrief. Findings in this scope feed into discussions on the facilitation of the solo practice itself. For instance, Campbell [29] found in her study, which involved students as participants, that a critical factor in determining the positive outcome of the solo was a positive pre-solo mindset. This is in line with the current study, where almost all participants expected the solo to be positive overall experiences, and reported a broad range of positively perceived well-being aspects.

Another theme stimulated by the results of this study is that of freedom vs. security. It stood out that most participants from the overall sample reported self-chosen low physical activity levels during solo. Such intuitive slow tempo may be important to the overall experience [69], enabling participants to more easily get in contact with the biodiversity surrounding them and create awareness for the stimulation of the different senses. However, about 50% of the participants from New Zealand, who were restricted in their physical space by the solo facilitators due to security reasons, mentioned perceived barriers due to a lack of physical movement. Campbell [29] saw similar tendencies in her study, where participants stated that physical restrictions during solo provoked psychological and physical pressure. In this sense, the current study supports the idea that the freedom to move freely can increase the chances to experience positivity perceived well-being-related outcomes.

The same might be valid for the possibility to choose a particular spot or environment for the solo. Expressed through the in vivo codes of this study, participants highlighted the importance of specific landscapes characteristics for their experiences. In the context of wilderness therapy, Nicholls [4] did not intend to focus on the role of the specific landscape environment, yet found its impact to be pervasive and extensive for the health-related outcomes of the participants. While some studies explicitly mention that some landscape, such as blue space, may hold more restorative potential than others [32], other studies point out that the aesthetic experiences elicited by wilderness landscapes are vital to an impact on well-being [70,71]. Interestingly, Richardson et al. [72] reported that aesthetic nature experiences also occur in urban environmental settings. Further, a recent paper found that engaging with nature was a more reliable factor in predicting and explaining variance in mental health and well-being than, for instance, the amount of time spent in nature [73]. This is in line with the findings of this study, which show that the reported range of well-being pathways seemed not to be compromised by solo duration. Consequently, shorter solos, for instance, in urban green areas [31] may also hold some potential for well-being.

### 4.7. Study Limitations, Strengths and Future Implications

The current study has four main limitations, which serve to inform future research topics and designs within the scope of solo. First, the overall comprehensiveness of the experienced well-being pathways could be compromised by applying a framework with pre-defined categories. Although having modified the basic PERMA framework and combining a concept- and data-driven analysis approach, it is possible that some well-being pathways remain uncovered. Nevertheless, the current study is arguably the first to examine such a broad range of well-being pathways resulting from nature solo experiences in one scope. Future research can build on the study’s findings to explore other possible well-being pathways and deepen the understanding of the proposed categories.

A related limitation is that a range of context variables have been identified to play an essential role in well-being-related outcomes (e.g., activation of senses, specific solo activities, landscape characteristics, the influence of weather). However, the investigation of these factors was a secondary exploration, and the study only provides a brief overview of these aspects. Consequently, this study can be considered a stepping stone for further inquiries exploring the contextual embedders of solo experiences, ranging from facilitation, landscape aspects, the impact of weather, specific activities during a solo, length, and the role of pre-experiences and expectations. Future research should also explore differences in well-being effects across the whole lifespan and deepen understanding of gender-related aspects.

In addition, the cross-sectional group debriefs of this study provided an opportunity to generate a broad overview of the range of well-being pathways. The fact that the debrief was group-based might have inspired participants to reflect on their experiences in multiple ways. However, when researching experiences, especially if linked to the investigation of emotions and other affective phenomena, challenges emerge regarding memory and privacy [74]. In-depth interviews in one-on-one settings could provide people with a more comfortable setting for speaking openly about their experiences. In this context, photo-elicitation has proven to support memory in the investigation of emotions within the outdoor context [22]. One may also consider interviewing experienced solo instructors or applying a longitudinal qualitative approach to explore the possible long-term well-being-related impact of solo experiences. The emergent list of well-being effects identified in this study may help to structure such interviews.

Lastly, one may question the overall transferability of the findings given the explorative nature of this study, the somewhat modest sample size, and the current sample of healthy (and mostly) younger adults. In this context, it is suggested that solo holds the potential to provide well-being-related outcomes in, for instance, therapeutic contexts. However, specific clinical samples would be needed to test such hypotheses. Moreover, the three presented national samples are embedded within Western, Educated, Industrialized, Rich, and Democratic (WEIRD) cultures. Nevertheless, WEIRD countries constitute only about 12% of the world population [75]. An important avenue for future research would be to investigate solo in other national samples, preferably from non-WEIRD cultures. In this scope, one may also consider applying a different framework since a cross-cultural comparison of the basic PERMA model of well-being could reveal that the model was a compromised fit for non-Western cultures [76]. The transdisciplinary GENIAL framework, recently proposed by Mead and Fisher [51], might, for instance, be a more appropriate choice since it reflects on the reciprocal relationships between multiple domains, levels of scale, and related social contextual factors that impact well-being.

## 5. Conclusions

So, why would a person voluntarily choose to isolate in nature? At least, in part, the answer is that solo experiences in nature offer a wide range of psychophysiological pathways to well-being. In the current study, pathways to hedonic and eudemonic well-being are represented by the six categories of PERMA-V: (Positive) emotions, engagement, relationships, meaning, accomplishment, and vitality. These well-being pathways showed to be highly interrelated and were, independently of gender and age, found in all three national samples. The study results suggest that universal and culture-specific aspects must be considered when investigating well-being pathways elicited by solo in nature. The findings also highlight how emotion and related affective phenomena are closely linked to all well-being pathways, particularly relationships. Interestingly, instead of experiencing loneliness, most participants reported increased connectedness to nature, themselves and others.

The secondary analysis of these well-being pathways shows that solo experiences are partly sensitive to their contextual situatedness, relating to the individual’s characteristics, the facilitation of the solo, and the nature of nature itself. For instance, prior solo experiences, the freedom to choose the landscape, and the weather can influence whether solo in nature feels like solitary confinement or is perceived as a positive, maybe life-changing, experience.

Given the steadily increasing interest in nature-based interventions [77], this study proposes solo as an unconventional but valuable experiential body-based practice beneficial to practical scopes beyond outdoor education, such as wilderness therapy and environmental or planetary health initiatives. Related to the COVID-19 pandemic, appreciation and interest for being in nature has increased among many [78,79]. At this moment in history, in particular, practising solo holds the potential to allay some of the anxiety and trauma associated with COVID-19 while nurturing kindness, creativity, and courage.

## Figures and Tables

**Table 1:** Participants and solo context characteristics for national samples: Norway, Germany, and New Zealand.

Characteristics		Norway	Germany	New Zealand (NZ)
**Participants**	Total (included in study)	14 (10 female, 4 male)	14 (8 female, 6 male)	12 (8 female, 4 male)
	age (average ± SD; range)	27 ± 7 (21–48 years)	25 ± 2 (22–29 years)	43 ± 6 (25–64 years)
	occupation	students	students	diverse occupations
	pre-experience with solo	9 (64%)	2 (14%)	4 (33%)
**Course** **context**	provided by	Norwegian University (voluntary participation)	German University (voluntary participation)	NZ outdoor education provider (voluntary participation)
	course embedment and aim	part of semester course, focus: *friluftsliv* (outdoor life), humans and nature	part of semester course, focus: from nature sports to outdoor experience)	part of a three-week outdoor education course, focus: personal growth
**Solo** **context**	landscape	mountains with lakes, far from next village	forest with stream, village close by	NZ bush, partly stream, far from next village
	choice of solo space	chosen by participants	chosen by participants	chosen by course instructor
	solo duration	48 h	24 h	60 h
	season/weather	spring/sunny	spring/sunny	late summer/sunny
	facilitation/priming (pre-brief, mid-visit, debrief)	instructor facilitated, pre-brief reflection task provided, no instructor mid-visit	instructor facilitated, pre-brief reflection task provided, no instructor mid-visit	instructor facilitated, reflection tasks provided, poem of Watts read before solo (see article intro), daily instructor visits

**Table 2:** The six well-being pathways by national sample, gender, age (younger vs. middle-aged) and prior solo experiences.

Well-Being Pathways (PERMA-V)	Combined Sample *n* = 40 (%)	Norway *n* = 14 (%)	Germany *n* = 14 (%)	New Zealand *n* = 12 (%)	Gender ♁; *n* = 26 ♂; *n* = 14	Age Y (21–44 Years; *n* = 31) M (45–64 Years; *n* = 9)	Prior Solo Experiences Yes (*n* = 15) No (*n* = 25)
**Emotions (and mood)**							
positively perceived	39 (98%)	13 (93%)	14 (100%)	12 (100%)	♁ (26); ♂ (13)	Y(30); M(9)	Yes (15); No (24)
negatively perceived	20 (50%)	8 (57%)	4 (29%)	8 (67%)	♁ (15); ♂ (5)	Y(15); M(5)	Yes (5); No (15)
**Engagement**							
perceived enablers	34 (85%)	11 (79%)	11 (79%)	12 (100%)	♁ (22); ♂ (12)	Y(26); M(8)	Yes (13); No (21)
perceived barriers	6 (15%)	0 (0%)	0 (0%)	6 (50%)	♁ (4); ♂ (2)	Y(3); M(3)	Yes (2); No (4)
**Vitality**							
positively perceived	33 (83%)	13 (93%)	8 (57%)	12 (100%)	♁ (21); ♂ (12)	Y(26); M(7)	Yes (14); No (19)
negatively perceived	9 (23%)	3 (21%)	0 (0%)	6 (50%)	♁ (6); ♂ (3)	Y(6); M(2)	Yes (2); No (7)
**Relationships**	38 (95%)	12 (86%)	14 (100%)	12 (100%)	♁ (25); ♂ (13)	Y(29); M(9)	Yes (14); No (24)
**Accomplishment**	27 (66%)	7 (50%)	10 (71%)	10 (83%)	♁ (18); ♂ (9)	Y(23); M(4)	Yes (7); No (20)
**Meaning**	21 (53%)	4 (29%)	11 (79%)	6 (50%)	♁ (11); ♂ (10)	Y(17); M(4)	Yes (5); No (16)

## Data Availability

The data presented in this study are available on request from the corresponding author. The data are not publicly available due to the sensitive information that the dataset contains.
